# Together Yet Apart: Remedies for Tensions Between Volunteers and Health Care Professionals in Inter-professional Collaboration

**DOI:** 10.1007/s11266-022-00492-5

**Published:** 2022-04-20

**Authors:** Georg von Schnurbein, Eva Hollenstein, Nicholas Arnold, Florian Liberatore

**Affiliations:** 1 Center for Philanthropy Studies (CEPS), University of Basel, Basel, Switzerland; 2 Swiss Centre for International Health, Swiss TPH, Basel, Switzerland; 3 Winterthur Institute of Health Economics (WIG), School of Management and Law, Zurich University of Applied Sciences, Zurich, Switzerland

**Keywords:** Inter-professional collaboration, Volunteer work, Health care provision, Inter-professional care, Volunteers, Dyadic perspectives, Volunteer coproduction

## Abstract

While volunteering is an essential factor in service delivery in many societal areas, the inclusion of volunteers in formal settings can also lead to tensions. In this article, we combine the literature on volunteering and inter-professional collaboration (IPC) to elaborate a framework regarding remedies for tensions between professional staff and volunteers within IPC in health care provision to ensure successful collaboration. Using a dyadic survey design to interview volunteers and volunteer managers, we show that the perspectives of volunteers and volunteer managers on the antecedents of effective IPC differ in paradoxical ways. While volunteer managers apply organizational logic concerning tasks and processes to avoid tensions, volunteers seek solutions on a relational basis. However, rather than trying to resolve these paradoxes, our study indicates that carefully managing tensions arising between volunteers and professional staff may be more successful than trying to resolve all tensions.

## Introduction

Volunteering is a frequent and well-respected specification of pro-social behavior, and volunteers are a core resource in many areas of social service, thus warranting particular attention. However, volunteering is not without its challenges. Especially in a formal setting, i.e., within organizations, volunteering induces several tensions, for instance, between paid staff and volunteers (Bittschi et al., 2019), voluntary appearance and operational planning (Cuskelly et al., 2006), qualification and empathy (Studer & von Schnurbein, 2013), and authenticity and formalism (la Cour, 2019). Tackling such tensions is critical for the future development of affected organizations and services (Smith & Lewis, 2011).

In this article, we take a closer look at volunteering in health and care services with a specific emphasis on tensions between volunteers and health care professionals (HCPs) and the underlying paradoxes. In many countries, the health and social care system is going through a period of severe pressure for better quality and increased efficiency. The government, service providers, and other stakeholder groups are searching for new concepts to tackle these challenges. One promising and widely respected concept is that of inter-professional collaboration (IPC). IPC occurs “when multiple health workers from different professional backgrounds provide comprehensive services by working with patients, their families, careers, and communities to deliver the highest quality of care across settings” (WHO, 2010, p. 13). The overarching aim of IPC is to increase the economic efficiency in this sector, as well as patient benefit and the satisfaction of the stakeholders involved (Zwarenstein et al., 2009). The systematic reviews of Karam et al. (2018) and Schot et al. (2020) show that effective IPC is characterized by (1) adequate organizational arrangements such as suitable information structures, clearly defined rules and responsibilities, and shared goals, as well as (2) an open and receptive professional culture including reciprocal trust, respect, mutual acquaintanceship, congruent philosophies and values, and a willingness to cooperate and communicate.

Although not listed in the definition above, volunteers may also be part of the collaborative teams in IPC (Reeves et al., 2018). Generally, volunteers fulfill an important function as coproducers in service delivery, providing effort, time, and information (Winter et al., 2019), which can be categorized into the following areas: socializing/recreational services (e.g., shared activities, emotional support), personal assistance (e.g., accompanying patients to the doctor, relieving care-giving family members for a few hours), administrative services (e.g., administrative support, communication and public relations), meal services, information services (e.g., orientation at hospital reception), group offers and counseling, or direct support of nursing professionals (e.g., on the hospital ward). The advantages of volunteer involvement in the health care sector comprise similar positive effects as in other sectors, such as social services or leisure, where studies report higher satisfaction with services (Studer, 2016) or higher net benefits (Hager & Brudney, 2004). Volunteers provide additional services that positively affect patient satisfaction, which cannot be performed by health care professionals due to staff shortages and missing reimbursement by tariff systems that only cover the costs related to medical services (Thomsen & Jensen, 2020). Additionally, volunteers offer an outside view and new perspectives on organizational activities, helping to prevent operational blindness (Rimes et al., 2017). Following the conceptual idea of IPC, volunteer work in health care comprises assisting health professionals in producing and delivering services (Nesbit et al., 2016) and enhances the quality of service provision (Studer & von Schnurbein, 2013). Different from other sectors with volunteer involvement, in the health care sector, the inclusion of volunteers is less criticized for taking over formally paid work for cost containment (Handy et al., 2007), compromising quality standards (Thomsen & Jensen, 2020), and covering failures in public service planning (Winter et al., 2019) because the work concerns nonmedical services that cannot be provided by health care professionals due to lack of time and resources. However, the IPC between paid staff and volunteers still bears the risk of task, process, relationship, and status conflicts between paid staff and volunteers, which are caused by manageable organizational conditions such as processes, structures, and task distribution (López-Cabrera et al., 2020). In the existing literature, the underlying paradox—understood as “contradictory yet interrelated elements that exist simultaneously and persist over time” (Smith & Lewis, 2011, p. 382)—with respect to the inclusion of volunteers in IPC is under researched, and there is a lack of explanations as to what extent and how tensions in the management of volunteers can be overcome. We thus aim to answer the following research questions:

1. What are antecedents of tensions between volunteers and HCPs in inter-professional health care provision?
2. Are there differences in the perspectives of volunteers and volunteer managers on the antecedents of tensions between volunteers and health professionals?

We propose to add to a better understanding of how tensions in volunteer management can be resolved or balanced in two ways. First, we combine the literature on volunteering and IPC to develop a framework for the management of volunteers in IPC. To date, the role of volunteers in IPC research has been largely neglected. However, as volunteers fulfill a special complementary function in health care service provision, their roles and interrelations within IPC with health professionals must be clarified. Second, research on the determinants of effective IPC relies mainly on conceptual considerations rather than empirical work. Referring to the complexity of inter-professional relationships, better knowledge of critical factors and their interrelations in reducing tensions and enhancing the quality of collaborative work is needed (Palanisamy et al., 2020; Schot et al., 2020). We test our literature-based hypotheses by applying a dyadic study design. The perspectives of volunteers and volunteer managers in health care organizations are solicited by standardized questionnaires on critical factors concerning the integration of volunteers and perceptions of efficient collaboration. Against the background of the complementary function of voluntary work in health care, and as some IPC studies find undesired and even adverse effects of a closer collaboration between health professionals in the health care provision (Lingard et al., 2017), the findings help to improve the understanding of volunteer management.

This article is structured as follows. Drawing on the literature on volunteering and IPC, we first develop a conceptual framework around the antecedents of tensions between volunteers and health professionals in IPC. Thereafter, we describe the study design and sample and present our findings with respect to remedies for tensions in inter-professional health care provision. Finally, we discuss our results and present practical recommendations.

## Conceptual Framework

Research on volunteer management is dominated by studies on the recruiting and retention of volunteers (Hager & Brudney, 2011; Kewes & Munsch, 2019), their qualification, and how to lead them (Kreutzer & Jäger, 2011). However, the understanding of the underlying logic of managing volunteers and potential tensions connected to it remains limited. Studies that analyze staff–volunteer relationships mostly focus on paid staff’s perspective on volunteers as a threat to their profession, service quality, and individual identity (Nesbit et al., 2016). In contrast, interorganizational tensions are a major factor for volunteers to leave the organization (Rimes et al., 2017). Only a few studies integrate the perspectives of organization managers on the question of volunteer management and how the paradox of combining paid and volunteer workforce can be solved. Kreutzer and Jäger (2011) build on the concept of organizational identity to show that tensions between volunteers and paid staff emerge from differentiations in authority, expectations, and motivation. Thomsen and Jensen (2020) separate core and complementary tasks in public service production. While volunteer work in the latter is accepted by paid staff, it is more likely to be seen as a threat in the former. Finally, Studer (2016) finds that specific leadership strategies may guarantee appropriate volunteer management and their successful integration in collaborative settings.

### Tensions in Staff–Volunteer Collaboration

Effective collaboration between volunteers and professional staff is key to providing high service quality. Volunteer placement must contribute to the efficient provision of services, and the benefits of their inclusion must exceed the costs of coordination, placement planning and, if necessary, qualification (Studer & von Schnurbein, 2013). Research on volunteers shows that poor volunteer–staff relationships are associated with outcomes such as decreased job satisfaction, stress and increased turnover intentions among both paid staff and volunteers (Rimes et al., 2017; Rogelberg et al., 2010). From the volunteer perspective, interpersonal relationships with other volunteers or paid staff in the organization are an important driver for continued engagement (Rimes et al., 2017). In contrast, paid staff members are more susceptible to tensions and report negative consequences of conflicts more often (López-Cabrera et al., 2020). Thomsen and Jensen (2020) differentiate between altruistic (quality concerns) and egoistic threats (concern for own tasks/own job), which may be perceived by paid staff in joint service provision. López-Cabrera et al. (2020) differentiate between conflicts of status, process, task, and relationship and identify fear of reprisals, frustration, and stress as the main consequences of conflicts on both sides. *Status conflicts* occur if paid staff perceive that volunteers are assigned to certain tasks that compromise the professional status and role of the paid staff. Hence, the perception of roles and appreciation of work may cause conflicts. In health care provision, many volunteer tasks are not core tasks according to the reimbursement systems but focus on increasing the well-being of patients. Listening to the needs and experiences of the patients by volunteers is a core part of caring. Consequently, health professionals may perceive a status conflict because they perceive these tasks as part of their professional role, which are now overtaken by volunteers (Thomsen & Jensen, 2020). *Process conflicts* address conflicts occurring during and along the joint service provision (IPC) due to role overlaps that disturb efficient workflows or are due to a lack of compliance of volunteers as a transgression of competences (López-Cabrera et al., 2020), which may endanger patient safety. *Task conflicts* evolve from different opinions about how to run specific tasks. Issues may include the communication of and the knowledge about tasks of both paid staff and volunteers, as well as the right prioritization of tasks throughout the health care provision. Paid staff may not understand what the volunteers are doing exactly and perceive them as intruders into the professional working environment, while volunteers may lack understanding of the complexity of professional health care. *Relationship conflicts* are defined by López-Cabrera et al., (2020, p. 4) as “personal incompatibilities, lacking recognition or tensions that provoke feelings such as frustration or irritation.” Avoidance is a common conflict management strategy to separate relationship conflicts and negative emotions between paid staff and volunteers (Benitez et al., 2018). Based on the identified types of conflicts and drawing on the literature on IPC and volunteers, in the following section, we present a framework for reducing tensions between paid staff and volunteers.

### Antecedents of Tensions Between Volunteers and Health Professionals in IPC

Within IPC research, D’Amour et al. (2005) reviewed different conceptual frameworks of collaboration. They conclude that all frameworks focus on the environment of collaboration, the processes of human interactions, and the resulting outcomes of IPC. Recent systematic reviews by Karam et al. (2018) and Schot et al. (2020) show that effective IPC is characterized by (1) adequate organizational arrangements such as a suitable information structure, clear rules and responsibilities, and shared goals and (2) an open and receptive professional culture including reciprocal trust, respect, mutual acquaintanceship, congruent philosophies and values, and a willingness to cooperate and communicate. Similarly, in the development of a measure to assess IPC, Nuño-Solinís et al. (2013) identify a two-factor structure comprising personal relationships between professionals and characteristics of the organizational environment. Palanisamy et al. (2020) stress the relevance of interactional determinants in addition to organizational structures for effective collaboration.

Research by Nesbit et al. (2018) on volunteer involvement in organizations shows that well‐defined organizational structures positively affect volunteers’ collaborative behavior and their experience in the organization and reduce role ambiguity. The structural characteristics include aspects of formalization, rules, and organizational decisions concerning volunteer roles. Rimes et al. (2017) identify divergent expectations, communication issues, behavioral or emotional discord, and perceptions of job vulnerability as critical mechanisms that foster volunteer–staff conflicts.

**Fig. 1:**
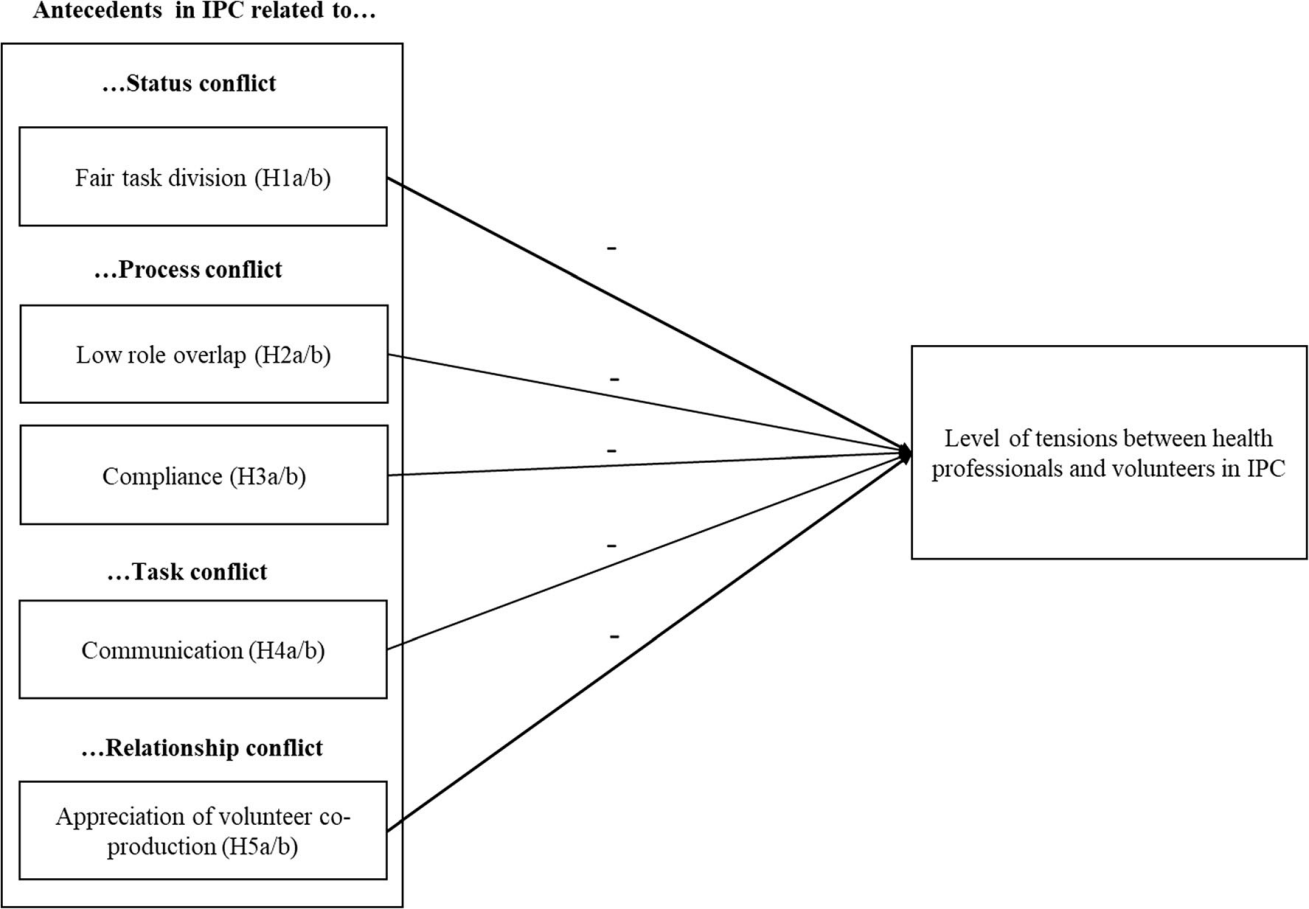
Antecedents of tensions between HCPs and volunteers

Figure 1 gives an overview of the antecedents of tensions between HCPs and volunteers, which have been identified in the literature on volunteering and in the literature on ICP. The rationale for the selection of these antecedents is explained below.

### Reduction of Status Conflicts Through Fair Task Division

Organizations employing volunteers face the challenge of defining and formalizing the tasks and roles of professional staff and volunteers within service provision, which is crucial for the reduction of tensions between volunteers and paid staff. If volunteer work is viewed as a substitute or replacement for paid work, status conflicts at the individual or professional group level are evoked (Thomsen & Jensen, 2020). From the perspective of paid staff, the mere perception of job vulnerability has the potential to incite volunteer–staff conflict (Merrell, 2000). Staff members often fear that volunteers may take jobs from them or that the use of volunteers will be cited as a reason for reducing an organization’s budget (Rimes et al., 2017). For volunteers, the relative role fit in the division of tasks between paid staff and volunteers has been associated with increased organizational identification and commitment by volunteers (Nesbit et al., 2018).

Despite the complementary nature of volunteer work in health care as an extra dimension to professional care, enriching and extending health care services, task division may be perceived as unfair by both sides. From HCPs’ perspective, volunteers partially perform elements of the core tasks of health provision (e.g., listening to the needs and experiences of the patients) that health professionals cannot provide to the patients due to time restrictions and missing reimbursement opportunities. From volunteers’ perspective, an instrumentalization of their engagement must be avoided. Furthermore, the tasks allocated to volunteers should be perceived as significant. Consequently, we hypothesize the following:

#### **H1a**

A fair and reasonable task division reduces tensions between volunteers and health professionals in IPC from the view of volunteer managers.

#### **H1b**

A fair and reasonable task division reduces tensions between volunteers and health professionals in IPC in the view of volunteers.

### Reduction of Process Conflicts Through Low Role Overlaps And Compliance

Role overlaps within IPC can create conflicts concerning responsibilities and task completion among health professionals. Therefore, negotiating overlaps has been identified as a major course of action to improve IPC (Schot et al., 2020). In the same vein, Karam et al. (2018) stress the importance of role clarification as an act of formalizing collaboration to enable IPC and to avoid confusion, power struggles and tensions on the organizational level.

Similarly, volunteer research detects appropriate task definitions between professionals and volunteers as critical for effective collaboration and for reducing process conflicts (López-Cabrera et al., 2020). An overlap of volunteers’ complementary activities with the core tasks of professionals should be avoided because overlaps counteract efficient and effective health care provision. Well-defined responsibilities and processes are recommended to reduce role ambiguity. However, a high level of formalization may also be perceived by volunteers as rigid and alienating and not fitting with their conception and aspirations regarding voluntary work (Nesbit et al., 2018). Nevertheless, clear tasks and role clarity have been identified as satisfaction-promoting volunteer job characteristics (Studer & von Schnurbein, 2013), reducing burnout and turnover (Nesbit et al., 2018). Therefore, we hypothesize the following:

#### **H2a**

A lower role overlap reduces the tensions between volunteers and health professionals in IPC from the view of volunteer managers.

#### **H2b**

A lower role overlap reduces the tensions between volunteers and health professionals in IPC in the view of volunteers.

While the formalization of volunteer roles in the organization is a critical structural factor, the compliance of volunteers with delegated tasks and responsibilities in service provision is a critical relational factor reducing tensions between volunteers and HCPs. In the health care sector, especially in interactions with vulnerable client groups, it is important that all persons involved in IPC adhere to their duties, obligations, and rights, especially because liability rules might otherwise be violated. However, volunteers in health care are often retired health professionals offering experiential knowledge and skills in the area of the core services of health care provision (Merrell, 2000). Therefore, they may be inclined to take over tasks from professional staff.

In IPC research, reciprocal trust and respect are relational factors enabling IPC (Karam et al., 2018). Being compliant with formal or negotiated agreements on the division of tasks is an important precondition for confidence building and respect within IPC. Where a formalization on the structural level within an organization is missing, relational trust may even be a substitute to guarantee effective IPC in health care provision (McDonald et al., 2012). However, in volunteer research, formal integration within an organization and the strict adherence to duties, obligations, and rights have been found to demotivate volunteers (Güntert et al., 2016; Studer & von Schnurbein, 2013). Volunteers prefer more adaptive structures (Nesbit et al., 2018), which are not always compatible with professional workflows and responsibilities. Tensions arise if professional staff question the use of volunteers because they fear that volunteers may undermine legal and professional standards (Taylor et al., 2006). In effect, López-Cabrera et al. (2020) identify the different views regarding duties and responsibilities as common sources of process conflict. We therefore hypothesize the following:

#### **H3a**

The compliance of volunteers with their roles and responsibilities has a direct and negative effect on tensions between volunteers and health professionals in IPC from the view of volunteer managers.

#### **H3b**

The compliance of volunteers with their roles and responsibilities has a direct and negative effect on tensions between volunteers and health professionals in IPC in the view of volunteers.

### Reduction of Task Conflicts Through Effective Communication

Effective communication is a crucial relational factor for reducing tensions in IPC. Communication facilitates the negotiation of ways of working, overcoming communicational divides, and creating a team culture, all of which have been identified as important ways in which professionals actively contribute to the effectiveness of IPC (Karam et al., 2018; Schot et al., 2020). Important personal relationship dimensions in IPC, such as trust, mutual knowledge and shared goals, are built by formal meetings and informal interpersonal interactions (Nuño-Solinís et al., 2013). Efficient, open, and equitable communication has been identified as an important mechanism for support and mutual acquaintanceship (Hewitt et al., 2014).

Volunteer research confirms that a lack of communication between volunteers and professionals is the most common source of conflicts and tensions in IPC (Nesbit et al., 2018). In their categorization, Rimes et al. (2017) highlight communication issues as one of four major sources of volunteer–staff conflicts. Communication deficiencies are responsible for antagonistic relationships between professionals and volunteers and hinder a sufficient acknowledgment of volunteer contributions (Studer & von Schnurbein, 2013). According to López-Cabrera et al. (2020), a lack of communication produces task conflicts and hinders effective coordination in joint service provision. We thus hypothesize the following:

#### **H4a**

 Regular communication between volunteers and health professionals reduces tensions between these actors in IPC in the view of volunteer managers.

#### **H4b**

Regular communication between volunteers and health professionals reduces tensions between these actors in IPC in the view of volunteers.

### Reduction of Relationship Conflict Through the Appreciation of Volunteer Coproduction

In IPC research, a lack of awareness of the other’s role in patient care has been defined as a major factor for distrust, which in turn negatively affects relational quality in IPC (Hewitt et al., 2014). Especially due to the dominance of health professionals in IPC with volunteers, mutual respect for joint contributions is a key element of balancing power and successfully working together (Karam et al., 2018).

In volunteer research, feeling unwanted and unappreciated as volunteer is an important reason for volunteers to leave an organization (Nesbit et al., 2018), as this produces relationship conflicts according to the typology of López-Cabrera et al. (2020). In turn, task significance, a variety of ways to contribute and sufficient acknowledgment by the organization are important satisfaction-promoting job characteristics for volunteers (Studer & von Schnurbein, 2013). According to the Swiss volunteer monitor (Freitag & Manatschal, 2016), volunteers value recognition for their work higher than financial incentives. Our final hypotheses are thus:

#### **H5a**

A greater appreciation of volunteer coproduction reduces tensions between volunteers and health professionals in IPC in the view of volunteer managers.

#### **H5b**

A greater appreciation of volunteer coproduction reduces tensions between volunteers and health professionals in IPC in the view of volunteers.

## Methods

Two standardized surveys were conducted, the first directed toward volunteer managers in Swiss health care organizations and the second administered to volunteers working in health care organizations in Switzerland. Both the conceptual framework and the surveys were part of a larger research project on the role of voluntary work within IPC in the Swiss health care system conducted on behalf of the Swiss Federal Office of Public Health. Switzerland is an appropriate setting for investigating the role of volunteers in IPC. First, voluntary work is carried out by approximately one-third of the Swiss population—amounting to approximately 700 million hours of unpaid work annually—and is an essential factor in the provision of services in many societal areas, including the health care sector (Bundesamt für Statistik, 2016; Freitag & Manatschal, 2016; Studer & von Schnurbein, 2013). Second, the Swiss health care system is increasingly applying IPC, and organizations are integrating an increasing number of volunteers in this area (Lamprecht et al., 2020).

### Questionnaire and Measures

Based on validated scales and questionnaire items from existing studies in the areas of volunteering and IPC (e.g.Freitag & Manatschal, 2016; Gentile et al., 2015; Rimes et al., 2017; Studer, 2016; Studer & von Schnurbein, 2013), two questionnaires were developed to test the framework presented above. The final elaboration of the questionnaires was aided by a critical review by eight experts who commanded either scientific knowledge of IPC and/or volunteerism within health care or expertise in how IPC with volunteers is experienced in practice within health care settings. The experts formed part of a Delphi group that was convened within the larger research project described above. In contrast to focus group discussions, in a Delphi group, participants are repeatedly involved in workshops to develop and evaluate project results (Niederberger & Renn, 2019).

The questionnaire directed at volunteer managers in health care institutions comprised 47 questions, while the questionnaire administered to volunteers included 39 questions. The main independent-level variables of interest (see Table 1)—fair task division as well as role overlap between professionals and volunteers, compliance of volunteers with respect to their roles and responsibilities, the degree of communication between professionals and volunteers, and appreciation of volunteer coproduction—appeared in similar forms in both questionnaires and were measured using a five-point Likert-scale format, with answers ranging from 1 = “does not apply” to 5 = “fully applies.” For the analysis of the antecedents for effective collaboration or tensions, we defined the mean value of the following items as dependent variables (see also: Table 1 in results section).

- *Questionnaire Volunteer Managers* “Tensions between volunteers and health professionals are uncommon;” “Our volunteers are often overstrained;” “Our health professionals generally perceive the inclusion of volunteers as positive” (reverse coded).
- *Questionnaire Volunteers* “I perceive tensions between volunteers and health professionals to be uncommon;” “I have often felt overstrained while volunteering;” and “I feel motivated in terms of my commitment” (reverse coded).

**Table 1:** Items descriptions and overview descriptive statistics (VS = volunteer survey, VMS = volunteer manager survey)

Dimension	Item in volunteer survey (VS) Item in organizational survey (VMS)	Mean (min = 1; max = 5)	Standard deviation
Fair task division	VS: I feel that the division of tasks between volunteers and paid staff is reasonable and fair	4.50	.713
	VMS: Our paid staff perceives the division of tasks between them and our volunteers as reasonable and fair	4.37	.683
Role overlap	VS: The tasks I perform overlaps with those of paid staff	2.54	1.150
	VMS: The tasks performed by our paid staff and our volunteers overlap	2.66	.841
Compliance	VS: I am aware of my duties, obligations, and rights	4.71	.536
	VMS: Our volunteers generally adhere to their duties and obligations	4.42	.572
Communication	VS: I can exchange information with paid staff on a regular basis	3.87	1.144
	VMS: Our paid staff and our volunteers exchange information on a regular basis	3.57	.945
Appreciation of volunteer coproduction	VS: The organization places great importance on utilizing my knowledge/skills	4.00	1.048
	VMS: We benefit greatly from the knowledge/skills that volunteers bring to our organization	3.70	.937
Items of the dependent variable: level of tensions	VS: I perceive tensions between volunteers and health professionals to be common	1.50	.707
	VMS: Tensions between volunteers and health professionals are common	1.79	.686
	VS: I have often felt overstrained while volunteering	1.48	.743
	VMS: Our volunteers are often overstrained	2.07	.835
	VS: I feel motivated in terms of my commitment	4.60	.650
	VMS: Our health professionals generally perceive the inclusion of volunteers as positive	4.42	.616

The reason for the use of a multi-item variable was to account for different facets of perceptions on effective collaboration or possible tensions as described in our conceptual considerations above. Perceived level of tensions, stress, and lack of motivation were identified as the main consequences of volunteer–staff conflicts in the study by López-Cabrera et al. (2020) for the absence of relationship, task and process conflicts between health professionals and volunteers, which are associated with complementary volunteer work designs in the López-Cabrera et al. (2020) typology and are geared to the items used in the study of Rogelberg et al. (2010).

### Data Collection and Analysis

Data collection took place in November and December 2019 and therefore was not influenced by the COVID-19 pandemic. In Switzerland, health care is one of the four major industries of the nonprofit sector (Helmig et al., 2017). The online survey directed at volunteer managers of health care institutions was sent to a total of 997 organizations from the German-, French- and Italian-speaking parts of Switzerland, including hospitals, nursing homes, physical rehabilitation centers, and relief organizations working in the health care sector (such as the Swiss Red Cross), and responded to by volunteer managers within the respective organizations. Volunteer managers are usually not health professionals. Their professional background is social work or a qualification in volunteer management. Either they are working part-time for the supervision, coordination and management of volunteers within the institutions, or this role is part of their task responsibilities in the social services department of a health provider. We have chosen volunteer managers due to the following reasons: First, due to their role and tasks for volunteer management they have an overall view on the conflicts and challenges in the inter-professional collaboration. Second, the view of health professionals about voluntary work would be very selective and incomplete. A total of 152 responses were obtained (response rate: 15.25%) from organizations, 127 (83.6%) of which used volunteers. Hence, the sample of volunteer managers in this study contains 127 answers from different organizations: 112 (88.2%) can be categorized as nursing homes, 22 (17.3%) each as organizations specializing in inpatient care and home care, and 6 (4.7%) as relief organizations (multiple answers possible). On average, these organizations employ 356 paid staff and 49 volunteers, with most volunteers (59.0%) active in the organization one or more times per week and for a period of more than 5 years (61.4%). Further details are provided in Appendix 1.

Regarding the questionnaire directed at volunteers, the health care organizations were asked to forward the survey link to volunteers working within their organizations, and a total of 318 responses were obtained. A total of 245 (77.0%) volunteer respondents were female, and most volunteers were close to or beyond retirement age (89.3%). The majority of volunteers were active in nursing homes (*n* = 130, 40.0%) or inpatient care organizations (*n* = 140, 44%), with most of them (67.5%) active in the organization once or more times per week and for a period of 2–5 years or more (79.2%). Further details are provided in Appendix 1.

For the empirical validation of our hypotheses, two separate multivariate regression analyses were conducted to investigate which variables from our conceptual considerations contribute to explaining the differences in the effective collaboration between volunteers and health professionals from the volunteer manager and volunteer perspectives, respectively.

## Results

### Conflict-Related Factors and Accompanying Tensions: Descriptive Information

Overall, 8.9% of volunteers confirmed the presence of tensions between volunteers and HCPs, and 8.3% confirmed that they felt overstrained while performing their tasks. Ninety-three percent of the volunteers felt strongly or rather strongly motivated in terms of their commitment. Considering motivation, perceived tensions and stress as different facets of consequences of conflicts, overall, approximately 8% of the volunteers in the sample are confronted with tensions.

Among responding volunteer managers in health care organizations, 23.8% at least partially support the statement “Our volunteers are often overstrained.” Furthermore, 11.6% indicate that tensions between volunteers and HCPs are at least partially common. A majority of 93.4% at least partially agreed with the statement “Our health professionals generally perceive the inclusion of volunteers as positive.” Overall, responding volunteer managers report the occurrence of tensions in their organizations to a higher extent than the volunteers. The descriptive statistics for all items are displayed in Table 1. Mean values for perceived minimum role overlap as a factor related to process conflicts, communication as a measure of reducing task conflicts, and appreciation of volunteer coproduction related to relationship conflicts show rather low values in the range of 2.0–4.0, whereas the mean value for the factors fair task division and compliance are in the range of 4.0–5.0 (see Table 1). The mean value of our multi-item-dependent variable indicating low levels of tensions between HCPs and volunteers was 4.55 in the volunteer survey and 4.18 in the volunteer manager survey.

### Antecedents of Tensions in IPC—Volunteer Manager Perspective

Table 2 shows the results of the regression analysis (*F* = 10.378, *p* = 0.000) based on the volunteer manager data sample. The variance inflation factors (VIFs) illustrate low multi-collinearity, and the adjusted *R*-square of 0.283 indicates moderate to good overall model fit. As suggested by Hair et al. (2010), the VIF should be below 5 to avoid a collinearity problem.

**Table 2:** Results of the regression on antecedents of tensions between volunteers and HCPs from the perspective of health care managers

Predictor variables	Nonstandardized B (SD)	Standardized (beta)	*t* value	*p* value	95% confidence interval		VIF
					Lower limit	Upper limit	
Constant	− 1.996 (.409)		− 4.876	.000***	3.192	4.815	
Fair task division (H1a)	− .153 (.063)	− .199	− 2.449	.016**	− .277	.029	1.098
Role overlap (H2a)	.099 (.050)	.159	1.992	.049**	.001	.197	1.057
Compliance (H3a)	− .274 (.077)	− .300	− 3.549	.001**	− .427	− .121	1.182
Communication (H4a)	− .109 (.051)	− .191	− 2.119	.036**	− .211	− .007	1.355
Appreciation of volunteer co-production (H5a)	− .049 (.049)	− .088	− 1.000	.320	− .146	.048	1.271
Corr. *R*^2^ = .283							

***p < .01; **p < .05

Generally, the dependent variable reports low or high perceived tensions between volunteers and paid staff. Accordingly, the significant negative beta-values for Hypotheses H1a, H3a, and H4a indicate that better task revision, compliance by volunteers, and better information exchange reduce such tensions. Additionally, Hypothesis H2a shows significant positive beta-values, e.g., less role overlap has a positive influence on the tensions. The perceived appreciation of volunteer coproduction does not have a significant effect on tension reduction, which is not significant according to the 95% confidence interval. Therefore, we reject Hypothesis H5a.

### Antecedents of Tensions in IPC—Volunteer’s Perspective

Table 3 shows the results of the regression analysis (*F* = 16.639, *p* = 0.000) based on the volunteer data sample. The variance inflation factors illustrate low multi-collinearity, and the adjusted *R*-square of 0.206 indicates moderate to good overall model fit (Hair et al. 2014).

**Table 3:** Results of the regression analysis on factors of tensions between volunteers and HCPs from the perspective of volunteers

Predictor variables	Nonstandardized B (SD)	Standardized (beta)	*t* value	*p* value	95% confidence interval		VIF
					Lower limit	Upper limit	
Constant	2.811 (.243)		11.551	.000***	2.332	3.290	
Fair task division (H1b)	− .139 (.040)	− .201	− 3.502	.001***	− .216	− 0.61	1.258
Role overlap (H2b)	.074 (.022)	.175	3.374	.001***	0.31	.117	1.023
Compliance (H3b)	− .086 (.053)	− .095	− 1.628	.105	− .189	.018	1.283
Communication (H4b)	− .048 (.026)	− .113	− 1.876	.062	− .099	.002	1.379
Appreciation of volunteer co-production (H5b)	− .084 (.029)	− .182	− 2.880	.004***	− .141	− .027	1.511
Corr. *R*^2^ = .206							

***p < .01; **p < .05

**Table 4:** Overview of the results

Nr	Hypotheses	Volunteer manager perspective	Volunteer perspective
	*Status conflict*		
H1a/b	Fair task division	Supported	Supported
	*Process conflicts*		
H2a/b	Low role overlap	Supported	Supported
H3a/b	Compliance	Supported	Not supported
	*Task conflict*		
H4a/b	Regular communication	Supported	Not supported
	*Relationship conflict*		
H5a/b	Appreciation of volunteer coproduction	Not supported	Supported

As in the volunteer manager sample, Hypotheses H1b and H4b show the same results that a fair task division and a low role overlap reduce perceived tensions. According to Hypothesis H5b, perceived appreciation of volunteer coproduction reduces tension perceptions by volunteers within IPC. The effect of the predictor variables compliance with their roles as volunteers as well as communication on the perceived occurrence of tensions between volunteers and HCPs from the perspective of volunteers exceed a significance level of *p* < 0.05 and are not significant according to the 95% confidence interval. We therefore reject Hypotheses H3b and H4b.

## Discussion and conclusion

Relying on volunteers while at the same time paying employees is a constant paradox of nonprofit management. The results of this study go beyond existing findings, especially by detecting remedies for tensions between volunteers and paid staff. Following the general research on paradoxes in management, our study of volunteers in IPC finds support for the fact that carefully managing tensions may be more successful than trying to resolve all tensions.

The descriptive results of this study confirm the findings of López-Cabrera et al. (2020) that in health care settings, conflicts related to processes, tasks and relationships are more frequent than those related to status. From the results of our study, a possible explanation for the reduced importance of status conflicts is the higher concentration of volunteers in complementary tasks, while paid staff executes the core services of health care provision. These findings do not differ in the dyadic samples of volunteers and volunteer managers. This result is contradictory to the results of López-Cabrera et al. (2020), where paid staff report conflicts to a higher extent than volunteers. However, they are in line with Rimes et al.’ (2017) results that volunteers and paid staff have comparable perceptions concerning the quality of volunteer–staff interactions. The inconsistent results highlight the necessity to further research the perceptions and judgments of the staff–volunteer relationship from different perspectives.

### Divergent Approaches to Reduce Tensions in Collaboration

One paradox of managing the collaboration of volunteers and paid staff is the difference in interactional and organizational logic (la Cour & Højlund, 2008). Volunteers engage because of their personal affection, and at the same time, they are embedded in an organizational structure. In our findings, the different antecedents to reduce tensions offer a reflection of this paradox. First, both sides, volunteers and volunteer managers, value fair task division and little role overlap as important criteria to reduce tensions. However, volunteer managers tend to focus on task- and process-related criteria such as improved communication and compliance between volunteers and paid staff for effective collaboration. In contrast, volunteers emphasize appreciation, a criterion of relationship, to make collaboration work. Hence, volunteer managers seek to integrate volunteers through managerial methods and instruments, while volunteers are more alert to relational aspects. As D’Amour et al. (2005) highlight, collaboration is based on collective action deduced from client needs and the development of a team life that integrates the different perspectives of volunteers and paid staff. This covers both aspects, organizational efforts to manage compliance with roles and initiate communication as well as building relations and show appreciation to the work of all actors involved in ICP.

### Navigating the Paradox in the IPC Setting

The second paradox we want to emphasize is related to the theoretical concept of IPC. As stated above, the aim of IPC is to increase the efficiency, client quality, and satisfaction of the actors involved through a more interrelated organization of health care. In our study, HCPs and volunteers in ICP preferred minimum role overlap and clear task division. More precisely, volunteers are assigned to complementary tasks, while HCPs—consisting of persons of different vocations and disciplines—are responsible for the core activities. Additionally, the tasks of volunteers and paid staff rarely take place simultaneously. More frequently, the tasks are in a serial order and not time-linked. Nevertheless, the tasks remain interdependent. Referring to the paradox of interactional and organizational logic of volunteer efforts, we argue that instead of aiming to solve the paradox, circumnavigation might be a more promising solution (Eliasoph, 2011). As Lingard et al. (2017) show, collaboration does not necessarily mean full integration. In their study, convergence and divergence coexisted simultaneously. Our study confirms this balanced approach: From an organizational logic, volunteers are integrated into an IPC setting with their separate roles and complementary tasks. Volunteer managers have to coordinate their participation and have to negotiate their integration in the overall schedule of the health care process. Instead of pushing for more collaboration, a loser structure of coaction is preferable (Schot et al., 2020). From an interactional logic perspective, volunteers and paid staff have to establish shared values and norms of how to provide health care services so that patients experience a coherent health care process.

### Limitations and Direction for Future Research

As is true with every study, ours also has some limitations. First, the study makes comparisons between volunteers’ and volunteer managers’ perspectives on tensions and related organizational and relational factors. Naturally, the view of volunteer managers may be limited to tensions that manifest themselves to them, neglecting tension that occurred between HCPs and volunteers, which were managed informally between the concerned individuals. Second, the data are based on a convenience sample because there are no complete data available on either volunteers or health care organizations in Switzerland. Therefore, the results may be subject to selection bias of experiences and personal characteristics of the participating volunteers and volunteer managers. Third, due to the relatively small sample size, the relevance of the antecedents of tensions could not be assessed in subsamples. Therefore, future studies should provide a differentiated analysis of tensions and conflict controlling for organizational, task and volunteer characteristics. Additionally, we call for in-depth analysis using qualitative data to better understand how tensions evolve and can be solved between volunteers and professionals. Given the complexity of health care, further research should elaborate on the recruitment, disposition, and retention of volunteers, especially in connection with the increase in episodic volunteering (Compion et al., 2022). Finally, future studies should use fluctuation rates or reported conflicts as indicators for tensions as outcome variables.

### Practical Implications

Finally, our study allows for some implications for the practice of volunteer management in health care organizations. For both groups, volunteers and paid staff, task and relationship conflicts are more frequent than status conflicts. Hence, volunteer managers should create an attitude of mutual appreciation, stimulate communication between volunteers and paid staff and assert compliance with the defined roles. Additionally, in consideration of the underlying paradoxes, the effective collaboration of volunteers and paid staff in an IPC setting will need both a balanced mix of integration in the inter-professional team and separated task provision of volunteers. The remedies for tensions detected in this study might help volunteer managers succeed in either solving tensions or circumnavigating the underlying paradox.

## Appendix 1: Sample Descriptions

**Table:**

Institutions (volunteer managers)	Frequency (*N* = 127)
*Organization type (multiple answers possible)*	
Inpatient medical care (hospitals, psychiatry and other crisis facilities, rehabilitation centers)	22 (17.3%)
Nursing homes	112 (88.2%)
Home care and accompanied living	22 (17.3%)
Relief organizations	6 (4.7%)
*Number of employees*	
Minimum	0
Maximum	10,750
Mean	356
Median	100
*Number of volunteers*	
Minimum	1
Maximum	800
Mean	49
Median	28
*Average frequency of deployment of individual volunteers*	
Once	0 (0.0%)
Irregular	39 (30.7%)
Once a month	13 (10.2%)
Once a week	60 (47.2%)
Multiple times a week	13 (10.2%)
N.A	2 (1.6%)
*Average length of stay of volunteers*	
Once	0 (0.0%)
Less than 1 year	0 (0.0%)
1–2 years	2 (1.6%)
2–5 years	37 (29.1%)
More than 5 years	78 (61.4%)
N.A	12 (9.4%)
*Total work hours (h) of all volunteers in institution*	
Minimum	50 h
Maximum	40′000 h
Mean	3637 h
Median	2000 h

Volunteers	Frequency (*N* = 318)
*Gender*	
Female	245 (77.0%)
Male	73 (23.0%)
*Age*	
15–24 years	1 (0.3%)
25–39 years	12 (3.8%)
40–54 years	21 (6.6%)
55–64 years	93 (29.2%)
65–74 years	150 (47.2%)
> 74 years	41 (12.9%)
*Education*	
No education completed	3 (0.9%)
Compulsory education	7 (2.2%)
Vocational training	138 (43.4%)
Diploma secondary school	23 (7.2%)
Higher technical and professional education	83 (26.1%)
Bachelor	25 (7.9%)
Master	30 (9.4%)
Others	9 (2.9%)
*Employment status*	
Full time	18 (5.6%)
Part time	63 (19.7%)
Unemployed/not working	205 (64.3%)
N.A	32 (10.0%)
*Organization type (multiple answers possible)*	
Inpatient medical care (hospitals, psychiatry and other crisis facilities, rehabilitation centers)	140 (44.0%)
Nursing homes	130 (40.9%)
Home care and accompanied living	40 (12.6%)
Relief organizations	22 (6.9%)
*Average frequency of deployment*	
Once	0 (0.0%)
Irregular	51 (16.1%)
Once a month	53 (16.7%)
Once a week	160 (50.5%)
Multiple times a week	53 (16.7%)
N.A	1 (0.3%)
*Average length of stay*	
Once	0 (0.0%)
Less than 1 year	26 (8.2%)
1–2 years	40 (12.6%)
2–5 years	92 (29.0%)
More than 5 years	159 (50.2%)
N.A	1 (0.3%)
